# EHPnet: NARSAD: The Mental Health Research Association

**Published:** 2007-08

**Authors:** Erin E. Dooley

In the early 1980s, three leading national mental health groups came together to form the American Schizophrenia Association. Since that time the organization has evolved in both name and scope; it is now known as NARSAD: The Mental Health Research Association to reflect its current focus on a wide range of mental disorders. NARSAD works not only to fund research to prevent, diagnose, and treat psychiatric diseases, but also to educate the public about these conditions. Visitors can find information on these initiatives and more at **http://www.narsad.org/**.

The Diseases and Conditions section of the NARSAD site is divided into six main sections: schizophrenia, depression, bipolar disorder, anxiety, childhood disorders, and other disorders. Each section offers a brief overview of the illness on which it focuses and a summary of symptoms and treatments. Insight into NARSAD’s research into the illness is also available. In addition, feature stories on related topics are provided, as is information for people recently diagnosed with the illness, including resources for learning about the illness they have been diagnosed with and the medications they have been prescribed.

The Research Center portion of the NARSAD site provides information for potential grant recipients on how to apply for funding, provides a list of grantees, and notifies visitors of NARSAD grant deadlines. A section of project summaries gives visitors a look at the work NARSAD supports. Among other projects are a twin study to tease out the nongenetic family background contributions to depression and a mouse study of how changes in maternal behavior associated with a genetic modification may be passed to offspring that do not possess the modification.

